# Rhein Ameliorates Cognitive Impairment in an APP/PS1 Transgenic Mouse Model of Alzheimer's Disease by Relieving Oxidative Stress through Activating the SIRT1/PGC-1*α* Pathway

**DOI:** 10.1155/2022/2524832

**Published:** 2022-03-22

**Authors:** Zhihui Yin, Demin Gao, Ke Du, Chen Han, Yuhan Liu, Ying Wang, Xiaoyan Gao

**Affiliations:** School of Chinese Materia Medica, Beijing University of Chinese Medicine, Beijing 102488, China

## Abstract

Mitochondrial oxidative stress plays an important role in the pathogenesis of Alzheimer's disease (AD). Recently, antioxidant therapy has been considered an effective strategy for the treatment of AD. Our previous work discovered that rhein relieved mitochondrial oxidative stress in *β*-amyloid (A*β*) oligomer-induced primary neurons by improving the sirtuin 1 (SIRT1)/peroxisome proliferator-activated receptor gamma coactivator 1-alpha- (PGC-1*α*-) regulated mitochondrial biogenesis. While encouraging results have been provided, mechanisms underlying the beneficial effect of rhein on AD are yet to be elucidated *in vivo*. In this study, we evaluated the therapeutic effect of rhein on an APP/PS1 transgenic (APP/PS1) mouse model of AD and explored its antioxidant mechanisms. As a result, rhein significantly reduced A*β* burden and neuroinflammation and eventually ameliorated cognitive impairment in APP/PS1 mice. Moreover, rhein reversed oxidative stress in the brain of APP/PS1 mice and protected neurons from oxidative stress-associated apoptosis. Further study revealed that rhein promoted mitochondrial biogenesis against oxidative stress by upregulating SIRT1 and its downstream PGC-1*α* as well as nuclear respiratory factor 1. Improved mitochondrial biogenesis not only increased the activity of superoxide dismutase to scavenge excess reactive oxygen species (ROS) but also repaired mitochondria by mitochondrial fusion to inhibit the production of ROS from the electron transport chain. Notably, the exposure of rhein in the brain analyzed by tissue distribution study indicated that rhein could permeate into the brain to exert its therapeutic effects. In conclusion, these findings drive rhein to serve as a promising therapeutic antioxidant for the treatment of AD. Our research highlights the therapeutic efficacy for AD through regulating mitochondrial biogenesis *via* the SIRT1/PGC-1*α* pathway.

## 1. Introduction

Alzheimer's disease (AD), the most prevalent progressive neurodegenerative disease, is characterized by cognitive impairment and memory decline [1]. Increasing evidence has shown that neuronal oxidative stress represents a crucial event in AD pathogenesis [2, 3]. Enzymes in mitochondrial electron transport chain (ETC) complexes, especially cytochrome c oxidase (CytOx) in complex IV, are devitalized by abnormally formed *β*-amyloid (A*β*) [4, 5]. Subsequently, electrons cannot be transported successfully, leading to electron leakage and high levels of reactive oxygen species (ROS), which give rise to oxidative stress in neurons along with decreased levels of catalase (CAT), glutathione peroxidase (GSH-Px), and glutathione (GSH) and increased levels of malondialdehyde (MDA) and oxidized GSH (GSSG) [6–8]. Seriously, excess ROS cause the oxidative damage of mitochondria and the release of cytochrome c from the mitochondrial membrane into the cytosol. As an apoptosis factor, cytochrome c activates the caspase 3-related apoptosis cascade and triggers the irreversible injury of neurons [9, 10]. Therefore, relieving mitochondrial oxidative stress is regarded as a promising therapeutic strategy for AD. Up to now, several antioxidants, including vitamin E [11], melatonin [12], and curcumin [13], have been considered candidates for the antioxidant therapy of AD. As these antioxidants relieve oxidative stress mainly through directly scavenging excessive ROS, limited therapeutic efficacy in AD patients has been obtained after treatment [14, 15]. Alternatively, modulating the redox homeostasis from an upstream perspective is a more effective strategy for long-term treatment of AD.

Mitochondrial biogenesis is considered an endogenous antioxidant defense system to maintain intracellular redox homeostasis [16, 17]. The sirtuin 1 (SIRT1)/peroxisome proliferator-activated receptor gamma coactivator 1-alpha (PGC-1*α*) pathway controls the mitochondrial biogenesis [18, 19]. SIRT1, a nicotinamide adenine dinucleotide-dependent histone deacetylase, is responsible for the deacetylation and activation of PGC-1*α*, a transcriptional coactivator. Then, PGC-1*α* targets downstream nuclear transcription factors, such as nuclear respiratory factor 1 (NRF1) and sequentially mitochondrial transcription factor A, which drives the transcription and replication of mitochondrial DNA (mtDNA) to generate healthy mitochondria [20]. The newly generated healthy mitochondria possessing high levels of antioxidant enzymes, principally superoxide dismutase (SOD), are sufficient to scavenge excessive ROS [21, 22]. In regard to damaged mitochondria, the newly generated healthy mitochondria can lessen them by a fusion process *via* mitochondrial dynamics to prevent the constant production of ROS from ETC in damaged mitochondria [23]. Regrettably, the SIRT1/PGC-1*α* pathway is blocked in AD pathology accompanied by the downregulated SIRT1 and PGC-1*α* as well as NRF1 [24, 25]. Thus, accumulated ROS cannot be eliminated, and damaged mitochondria cannot be repaired, ultimately neuronal apoptosis. Therefore, the SIRT1/PGC-1*α* pathway is the key component in controlling endogenous antioxidant function and resisting oxidative stress in AD. Activation of the SIRT1/PGC-1*α* pathway to improve mitochondrial biogenesis has potential for the treatment of AD.

Rhein (4,5-dihydroxyanthraquinone-2-carboxylic acid), a type of anthraquinone compound derived from rhubarb, is identified as a potent antioxidant for multiple diseases, such as cerebral ischemic/reperfusion [26], hyperglycemia [27], and traumatic brain injury [28]. By establishing an A*β*_42_ oligomer-burdened neuron model, our previous work has proven that rhein notably mitigated intracellular ROS levels, reversed the depletion of mitochondrial membrane potential in neurons, and protected neurons from mitochondrial oxidative damage-associated apoptosis [29]. Moreover, activation of the SIRT1/PGC-1*α*-regulated mitochondrial biogenesis is an essential mechanism of rhein for mobilizing the mitochondrial antioxidant defense system. However, the therapeutic evaluation and antioxidant mechanisms of rhein for AD remain to be verified *in vivo*.

In the present study, we examined the antioxidant effect and mechanisms of rhein on APP/PS1 transgenic (APP/PS1) mice. Our results demonstrated that a one-month treatment of rhein improved the pathological phenotypes and cognitive function in APP/PS1 mice. Furthermore, rhein played a role in preventing neurons from oxidative damage-associated apoptosis. Most importantly, rhein relieved oxidative stress in the brain of APP/PS1 mice by promoting the SIRT1/PGC-1*α*-regulated mitochondrial biogenesis as an endogenous antioxidant defense system. Mitochondrial biogenesis not only afforded endogenous antioxidant enzymes against oxidative stress but repaired damaged mitochondria *via* the mitochondrial fusion process to inhibit the production of ROS from an upstream perspective. Moreover, the exposure of rhein in the brain was confirmed by pharmacokinetic and brain distribution studies. From the above, rhein could be a potential therapeutic agent for AD.

## 2. Materials and Methods

### 2.1. Animals and Treatments

Six-month-old male APP/PS1 mice and age-matched wild-type (WT) mice with the same background (C57BL/6) were obtained from Junke Biological Co., Ltd. (Nanjing, China). Sprague-Dawley (SD) rats and ICR mice were obtained from SPF Biotechnology Co., Ltd. (Beijing, China). All rats and mice were housed with a 12-hour light/dark cycle, constant temperature (25 ± 2°C), and relative humidity of 60 ± 5%. Among them, APP/PS1 and WT mice were housed one mouse per cage. Standard chow diet and water were allowed ad libitum. All experimental procedures were approved by the Ethics Committee for Animal Care and Treatment of Beijing University of Chinese Medicine. Animal suffering and the number of animals used were minimized to the great extent.

Rhein (CAS: 478-43-3, purity > 98%) was purchased from Shanghai Aladdin Biochemical Technology Co., Ltd. (Shanghai, China). APP/PS1 and WT mice were randomly divided into three groups: the WT group (*n* = 6), the APP/PS1 group (*n* = 6), and the APP/PS1+rhein group (*n* = 6). They were intravenously injected with PBS, PBS, and rhein (20 mg/kg body weight) every two days for one month, respectively. In the third week during treatment, behavior tests were performed. After one-month treatment, the mice in different groups were sacrificed for therapeutic evaluation.

### 2.2. Pharmacokinetic and Brain Distribution Studies

#### 2.2.1. Plasma Collection

Five SD rats were used for pharmacokinetic study. After a single intravenous injection with rhein (10 mg/kg body weight), blood samples (0.3 mL) were collected from the orbital venous plexus before and 0.083, 0.25, 0.5, 1, 2, 4, 6, 8, 12, and 24 h after administration in heparinized tubes. The blood samples were centrifuged at 4°C (3500 rpm, 10 min) to obtain plasma.

#### 2.2.2. Brain Tissue Collection

Fifteen ICR mice were used for brain distribution study. After a single intravenous injection with rhein (10 mg/kg body weight), ICR mice were euthanized followed by being perfused with saline at 1, 4, and 12 h, respectively (*n* = 5 per time point). Then, the brains were collected, washed with saline, blotted dry with filter paper, and accurately weighed. After that, the brains were homogenized in cold saline to obtain homogenates.

The concentrations of rhein in plasma and brain homogenates were analyzed by ultraperformance liquid chromatography coupled with triple quadrupole mass spectrometry (UPLC-TQ-MS/MS) (see Supplementary Materials).

#### 2.2.3. Pharmacokinetic Parameter Analysis

The pharmacokinetic parameters, including elimination half-life (*t*_1/2_), area under the plasma concentration-time curve from time 0 to the last time (AUC_0‐*t*_), area under the plasma concentration-time curve from time 0 extrapolated to infinite time (AUC_0‐∞_), mean residence time (MRT), apparent volume of distribution (*V*_d_), and clearance (CL), were analyzed by noncompartmental analysis using WinNonlin 8.1 (Certara, Princeton, NJ, USA).

### 2.3. Behavior Tests

#### 2.3.1. Nesting Test

Nesting test was performed for three days. On the first day, 10 pieces of paper (5 × 5 cm^2^) were placed per cage for nesting. The nesting behavior was recorded every day by taking photos. On the third day, the nesting score was evaluated by the 4-point system [30].

#### 2.3.2. Novel Object Recognition (NOR) Test

The NOR test was performed in an arena of 45 × 45 × 66 cm^3^ as described previously [31]. Before the test, the arena and objects (cubes and cylinders) were cleaned with 75% ethanol to fully remove the smell. On the first day, each mouse was placed into the arena for 30 min to adapt to the environment. On the second day, two familiar objects (cubes) were placed in the box and each mouse was allowed to familiarize for 5 min. On the third day, one of the two familiar objects was replaced by the novel one (cylinder), which was different from the familiar object in shape, size, and color. Then, the mouse was placed into the arena to explore for another 5 min. Their behaviors were recorded and analyzed by a video tracking system (Noldus Information Technology Co., Ltd, Wageningen, Netherlands). The time spent on the exploration of familiar/novel objects was calculated to evaluate the exploration and memory abilities.

### 2.4. Immunohistochemistry (IHC)

The whole brains were harvested and fixed in 10% formalin. The brains were embedded in paraffin and sectioned at 10 *μ*m. Then, the sections were washed thrice with PBST and blocked with bovine serum albumin working solution for 30 min at room temperature. After that, the sections were incubated with anti-A*β* antibody or anti-Iba-1 antibody (Abcam, Cambridge, MA, USA) for 4 h at 37°C, followed by being incubated with a secondary antibody for 40 min at 37°C. After being stained with diaminobenzidine, the images for IHC were taken under a fluorescence microscope (Nikon Instruments Inc., Melville, NY, USA).

### 2.5. Measurement of A*β*_42_, TNF-*α*, and IL-1*β*

The hippocampal tissues were homogenized in ice-cold lysis buffer, followed by being centrifuged at 4°C (10000 × *g*, 5 min). The supernatant was collected as the fraction of soluble A*β*_42_, tumor necrosis factor-*α* (TNF-*α*), and interleukin-1*β* (IL-1*β*). The pellets were washed with ice-cold lysis buffer three times to fully remove soluble A*β*_42_, and the final pellets were collected as the fraction of insoluble A*β*_42_. After being quantified by the bicinchoninic acid (BCA) assay (Beyotime Biotechnology, Shanghai, China) and adjusted to the same concentration, the levels of soluble A*β*_42_, insoluble A*β*_42_, TNF-*α*, and IL-1*β* were measured by using different ELISA kits (Cloud-Clone Corporation, Wuhan, China) according to the corresponding manufacturer's instructions.

### 2.6. Nissl Staining

The whole brains were harvested and fixed in 10% formalin and embedded in paraffin. Then, the embedded brains were sectioned and stained with Nissl staining solution for the observation of the Nissl body in the neurons of the cortex and hippocampus. Finally, the images of Nissl staining sections were taken under a fluorescence microscope.

### 2.7. Measurement of MDA, CAT, GSH-Px, GSH, GSSG, SOD, and CytOx

The hippocampal tissues were homogenized in ice-cold lysis buffer, and the supernatant was collected after centrifugation. After being quantified by the BCA assay, the levels of MDA, CAT, GSH-Px, GSH, GSSG, and SOD were measured by using detection kits obtained from Beyotime Biotechnology (Shanghai, China), and the activity of CytOx was measured by using a detection kit obtained from Solarbio Science & Technology Co., Ltd. (Beijing, China) according to the corresponding manufacturer's instructions.

### 2.8. Western Blot

The hippocampal tissues were homogenized in ice-cold lysis buffer containing 1 mM PMSF for protein extraction. Protein samples were quantified, separated by SDS-PAGE, and transferred onto polyvinylidene fluoride membranes. After being blocked with TBST containing 5% nonfat milk, the membranes were incubated at 4°C overnight with the following primary antibodies, including cytochrome c, caspase-3, PGC-1*α*, NRF1, mitofusin 1 (MFN1), and *β*-actin obtained from Proteintech (Rosemont, IL, USA), dynamin-related protein 1 (DRP1), and SIRT1 obtained from Cell Signaling Technology Inc. (Danvers, MA, USA). The membranes were incubated with secondary antibodies for 2 h at room temperature, soaked with ECL Chemiluminescent Substrate, and visualized by using a ChemiDoc™ MP Imaging System (Bio-Rad Laboratories, Inc., Hercules, CA, USA). The results were presented in relative protein levels after normalization to *β*-actin.

### 2.9. Statistical Analysis

The results are expressed as the mean ± standard deviation (SD). Data analysis was performed using SPSS Statistics 25.0 (IBM, Armonk, NY, USA). One-way analysis of variance (ANOVA) was used for the statistical significance analysis. Statistical significance was accepted at *p* < 0.05.

## 3. Results and Discussion

### 3.1. Pharmacokinetic Properties and Brain Distribution of Rhein

**Figure 1:**
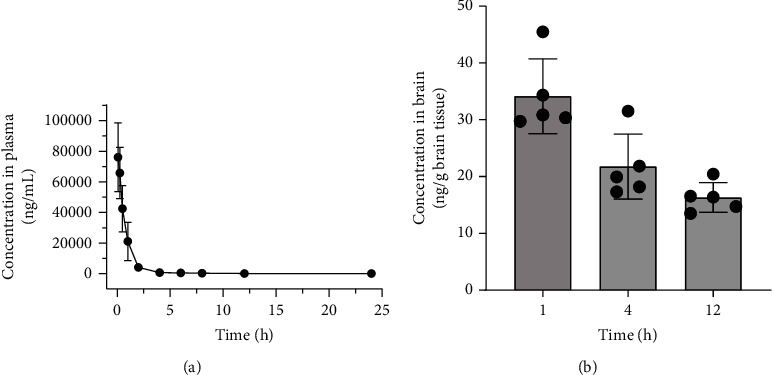
Pharmacokinetics profiles of rhein. (a) Concentration-time curve of rhein in plasma at 0, 0.083, 0.25, 0.5, 1, 2, 4, 6, 8, 12, and 24 h after a single intravenous injection with rhein (*n* = 5). (b) Brain distribution of rhein at 1, 4, and 12 h after a single intravenous injection with rhein (*n* = 5).

In the beginning, the pharmacokinetics of rhein was investigated to clarify its fate *in vivo*. The mean concentration-time curve is illustrated in Figure 1(a). After a single intravenous injection to SD rats, the concentrations of rhein in the plasma decreased rapidly within the first one hour, followed by a relatively gentle elimination until the last 24 h. The fitting main pharmacokinetic parameters are listed in Table 1. The *t*_1/2_, AUC_0‐*t*_, MRT, and CL were 8.09 h, 60.42 *μ*g·h/mL, 1.47 h, and 0.16 L/h/kg, respectively. It was shown that rhein exhibited a rapid clearance in the plasma of rats.

Next, the exposure of rhein in the brain of ICR mice was analyzed and is shown in Figure 1(b). In the first hour, 34.15 ng/g brain tissue of rhein was detected with the maximum concentration, showing that it quickly accumulated in the brain through the blood circulation. And then, reduced concentrations were found at 4 and 12 h, indicating its clearance from the brain. The above results demonstrated that rhein could cross the blood-brain barrier (BBB) and permeate into the brain to exert its therapeutic effects for AD.

### 3.2. The Therapeutic Evaluation of Rhein in APP/PS1 Mice

**Figure 2:**
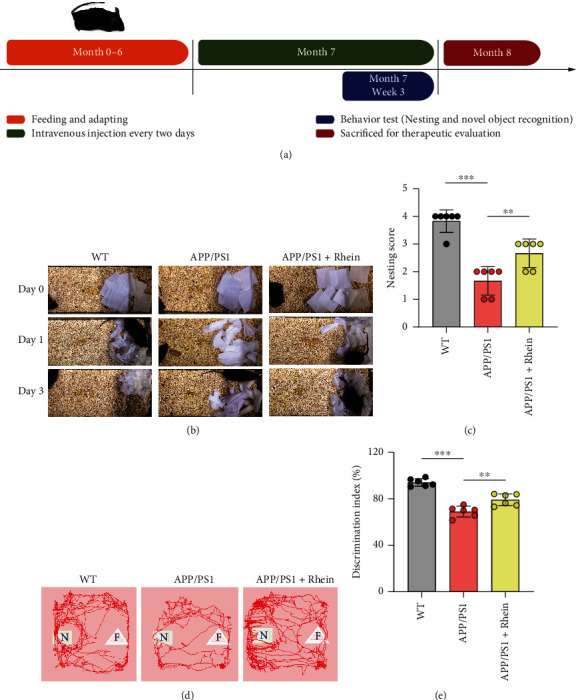
Rhein ameliorated cognitive impairment in APP/PS1 mice after a one-month treatment course by intravenous injection. (a) Time schedule of the experimental procedure. Behavior evaluation was performed in APP/PS1 mice and wild-type (WT) mice by the nesting and novel object recognition (NOR) tests. (b) Representative images from 0-3 days during the nesting test and (c) quantitative analysis of nesting behavior in day 3 (*n* = 6). (d) Representative tracing images and (e) quantitative analysis of exploration and memory abilities during the NOR test (*n* = 6). N: novel objects; F: familiar objects. The results are expressed as the mean ± standard deviation (SD). ^∗∗^*p* < 0.01, ^∗∗∗^*p* < 0.001 compared with the APP/PS1 group.

In this study, six-month-old APP/PS1 mice and age-matched WT mice were used to evaluate the therapeutic effect of rhein *in vivo*. The time schedule is illustrated in Figure 2(a). Briefly, rhein was intravenously injected into mice every two days for one month, and behavior tests including nesting and NOR were performed in the third week simultaneously. After one-month treatment, the mice in different groups were sacrificed for therapeutic evaluation.

#### 3.2.1. Rhein Ameliorated Cognitive Impairment in APP/PS1 Mice

In behavior tests, the nesting test was used to assess the cognitive ability of APP/PS1 mice. As illustrated in Figure 2(b), there were no identifiable nest sites or noticeable tearing of the paper in the APP/PS1 group, thus resulting in the lowest nesting score (Figure 2(c)). It indicated a cognitive decline in APP/PS1 mice. Interestingly, rhein treatment mitigated the cognitive decline of APP/PS1 mice according to the obvious nest behavior and increased nesting score. The NOR test was used to estimate the exploration and memory abilities of APP/PS1 mice. Compared with WT mice, APP/PS1 mice spent less time exploring the novel objects, suggesting that APP/PS1 mice could not discriminate the familiar (F) and novel (N) objects because of exploration and memory deficits (Figures 2(d) and 2(e)). Impressively, rhein increased the discrimination for novel objects. All these results demonstrated that rhein could significantly ameliorate cognitive and memory impairment in APP/PS1 mice.

#### 3.2.2. Rhein Reduced A*β* Burden and Neuroinflammation in APP/PS1 Mice

**Figure 3:**
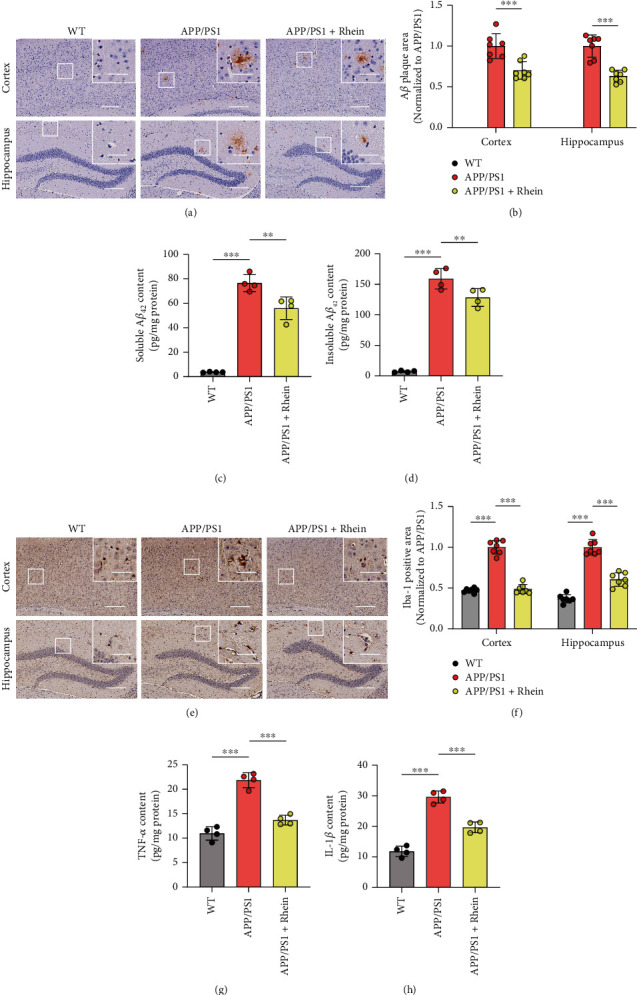
Rhein reduced A*β* burden and neuroinflammation in APP/PS1 mice. (a) Immunohistochemical staining and (b) quantitative analysis of A*β* plaques in the cortical and hippocampal regions (*n* = 7). Scale bar: 200 *μ*m. The images in the upper right corner showed the magnified regions in white boxes. Scale bar: 50 *μ*m. (c, d) Quantification of soluble and insoluble A*β*_42_ contents in the hippocampus by using ELISA kits (*n* = 4). (e) Immunohistochemical staining and (f) quantitative analysis of Iba-1 for reactive microglial activation in the cortical and hippocampal regions (*n* = 7). Scale bar: 200 *μ*m. The images in the upper right corner showed the magnified regions in white boxes. Scale bar: 50 *μ*m. (g, h) Quantification of TNF-*α* and IL-1*β* contents in the hippocampus by using ELISA kits (*n* = 4). The results are expressed as the mean ± standard deviation (SD). ^∗∗^*p* < 0.01, ^∗∗∗^*p* < 0.001 compared with the APP/PS1 group.

A*β* deposits in the brain are a curial pathological phenotype in AD [32–34]. To evaluate the therapeutic effect of rhein on APP/PS1 mice, A*β* plaques in the cortex and hippocampus were detected by immunohistochemistry. As depicted in Figures 3(a) and 3(b), A*β* deposits were not detected in the cortex or hippocampus of WT mice, but a distinct plaque area and number could be found in APP/PS1 mice. In contrast, rhein treatment markedly reduced the A*β* plaques in the brain of APP/PS1 mice. As the hippocampus is an important region in the brain responsible for learning and memory storage [35], the accurate A*β*_42_ content in the hippocampus was further measured. As quantified by using ELISA kits (Figures 3(c) and 3(d)), the amounts of soluble and insoluble A*β*_42_ in the hippocampal tissues of APP/PS1 mice were clearly reduced in rhein-treated APP/PS1 mice compared with those without treatment.

Sustaining reactive microglial activation induces chronic neuroinflammation through secreting proinflammatory factors, which dominate another curial pathological phenotype of AD [36, 37]. Iba-1 immunofluorescence staining was further adopted to detect activated microglia in the cortex and hippocampus. Compared with WT mice, a significant increase in Iba-1-positive microglia was observed in the cortical and hippocampal regions of APP/PS1 mice (Figures 3(e) and 3(f)). Interestingly, an apparent reduction of Iba-1 immunoreactivity in these regions was observed after treatment with rhein. In addition, to further verify the effect of rhein on neuroinflammation, two proinflammatory factors, TNF-*α* and IL-1*β*, in the hippocampal tissues were measured by using ELISA kits. The levels of TNF-*α* and IL-1*β* in APP/PS1 mice were 2 and 3 times higher than those in WT mice, respectively (Figures 3(g) and 3(h)). In contrast, following continuous treatment with rhein, both of them were markedly reduced, which was consistent with the results of immunofluorescence staining.

According to the behavior tests, histopathology, and pathophysiological results, the ability of rhein to alleviate the pathological phenotypes and cognitive impairment in AD was positively suggested.

### 3.3. Rhein Reversed Neuronal Degeneration by Relieving Oxidative Stress in the Brain of APP/PS1 Mice

**Figure 4:**
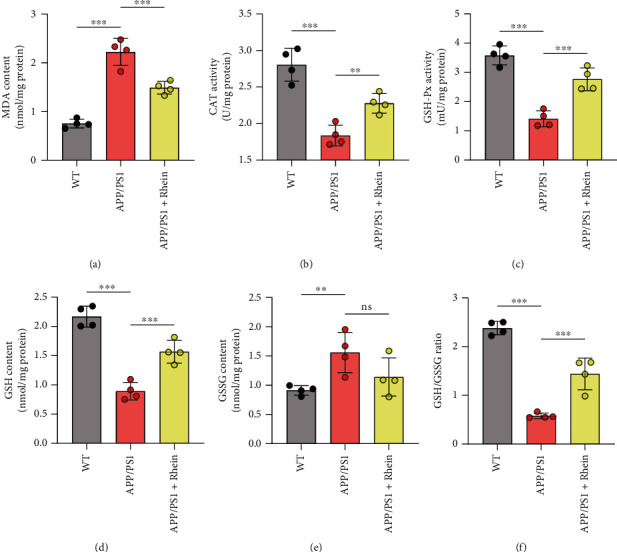
Rhein alleviated oxidative stress in the hippocampus of APP/PS1 mice. (a–f) Various oxidative stress-related biomarkers, MDA content (*n* = 4), CAT activity (*n* = 4), GSH-Px activity (*n* = 4), GSH content (*n* = 4), GSSG content (*n* = 4), and GSH/GSSG ratio (*n* = 4), were assessed using biochemical assay kits. The results are expressed as the mean ± standard deviation (SD). ^∗∗^*p* < 0.01, ^∗∗∗^*p* < 0.001. ns: not significant compared with the APP/PS1 group.

Our previous research showed that rhein effectively relieved oxidative stress in primary neurons induced by A*β*_42_ oligomers. Herein, the oxidative stress-related biomarkers in the hippocampus of the brain were detected to evaluate the antioxidant activity of rhein *in vivo*. Increased MDA represents lipid peroxide, which is toxic to neurons [38]. Indeed, there was a pronounced high level of MDA in APP/PS1 mice compared with WT mice, implying the oxidative damage in the brain (Figure 4(a)). Notably, rhein decreased the MDA content in APP/PS1 mice. Excess ROS exceedingly consume endogenous antioxidant enzymes, such as CAT and GSH-Px, bringing out lower activities of enzymes [39]. As expected, compared with the WT group, the activities of CAT and GSH-Px were both decreased in APP/PS1 mice (Figures 4(b) and 4(c)). Similarly, rhein restored this adverse situation. In addition, lower GSH level and higher GSSG level indicate a weak oxidation resistance [40], which appeared in the deceased GSH content and GSH/GSSG ratio, and increased GSSG content in APP/PS1 mice (Figures 4(d)–4(f)). In contrast, rhein remarkably increased the GSH content and GSH/GSSG ratio, although there was no significant decrease in GSSG content compared with the APP/PS1 group. Accordingly, it could be concluded that rhein was effective in relieving oxidative damage in the brain of APP/PS1 mice.

**Figure 5:**
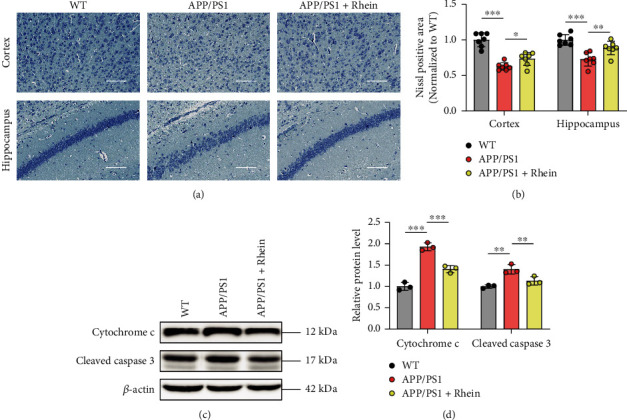
Rhein reversed neuronal degeneration in APP/PS1 mice by relieving oxidative stress. (a) Nissl staining and (b) quantitative Nissl-positive areas in the cortical and hippocampal regions (*n* = 7). Scale bar: 100 *μ*m. (c) Representative western blot bands of cytosolic cytochrome c and cleaved caspase-3 in the hippocampus. (d) Quantitative analysis of cytosolic cytochrome c and cleaved caspase-3 by normalization to *β*-actin (*n* = 3). The results are expressed as the mean ± standard deviation (SD). ^∗^*p* < 0.05, ^∗∗^*p* < 0.01, and ^∗∗∗^*p* < 0.001 compared with the APP/PS1 group.

Neurons are particularly vulnerable to oxidative stress, and neuronal oxidative damage is considered a key component in AD pathogenesis [16, 41]. Indeed, Nissl staining indicated that the cortex and hippocampus of APP/PS1 mice expressed the lower density and number of neurons compared with WT mice, reflecting a typical neuronal injury (Figures 5(a) and 5(b)). However, after treatment with rhein, a Nissl-positive area was clearly increased, which indicated the neuroprotection of rhein. Recent studies have reported that neuronal oxidative stress destroys mitochondrial membrane permeability and thus causes the release of cytochrome c into the cytosol from mitochondria. Cytosolic cytochrome c activates procaspase-3 to cleaved caspase-3, subsequently resulting in neuronal apoptosis [9, 10]. In this aspect, neuronal apoptosis-related protein expression levels of cytosolic cytochrome c and cleaved caspase-3 in the hippocampal tissues of APP/PS1 mice were further analyzed by western blotting. As shown in Figures 5(c) and 5(d), compared with WT mice, the expression level of cytosolic cytochrome c in APP/PS1 mice was much higher. Hence, the high level of cytosolic cytochrome c upregulated the protein level of cleaved caspase-3. After treatment with rhein, both the levels of them were significantly decreased. The above results provided evidence that rhein had an effect on neuroprotection by inhibiting oxidative damage-associated neuronal apoptosis.

### 3.4. Rhein Improved the SIRT1/PGC-1*α*-Regulated Mitochondrial Biogenesis

**Figure 6:**
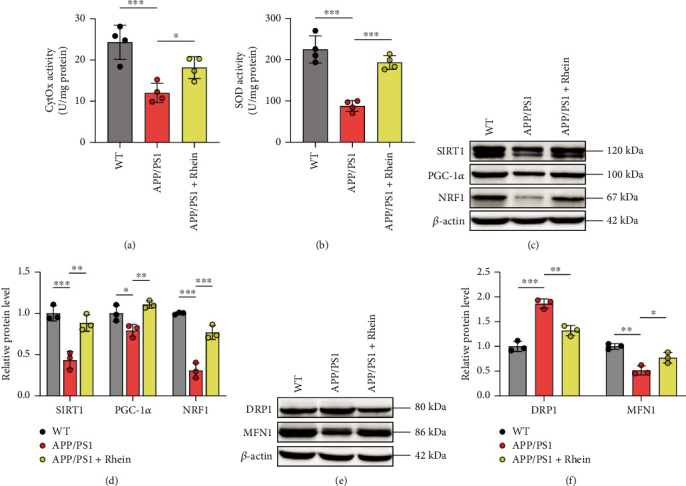
Rhein improved mitochondrial biogenesis to overcome neuronal oxidative stress by activating the SIRT1/PGC-1*α* pathway. (a, b) The activities of mitochondrial cytochrome c oxidase (CytOx) and superoxide dismutase (SOD) in the hippocampus (*n* = 4). (c) Representative western blot bands of SIRT1, PGC-1*α*, and NRF1 in the hippocampus. (d) Quantitative analysis of SIRT1, PGC-1*α*, and NRF1 by normalization to *β*-actin (*n* = 3). (e) Representative western blot bands of DRP1 and MFN1 in the hippocampus. (f) Quantitative analysis of DRP1 and MFN1 by normalization to *β*-actin (*n* = 3). The results are expressed as the mean ± standard deviation (SD). ^∗^*p* < 0.05, ^∗∗^*p* < 0.01, and ^∗∗∗^*p* < 0.001 compared with the APP/PS1 group.

Mitochondria are the main production place of ROS, and abnormal mitochondrial function occurs in AD [2]. Toxic A*β* decreases the activities of enzymes (such as CytOx) in ETC complexes and destroys ETC on the mitochondrial inner membrane, thus leading to a high level of ROS [4]. Indeed, the activity of CytOx in the mitochondrial ETC complex IV was decreased in APP/PS1 mice (Figure 6(a)). Given that rhein could effectively alleviate oxidative stress *in vivo*, we speculate that rhein relieved oxidative stress through modulation of mitochondria. As expected, after treatment with rhein, CytOx significantly recovered its activity. Additionally, rhein restored the activity of SOD, a mitochondria-derived antioxidant enzyme (Figure 6(b)). Preliminary results manifested that rhein could relieve oxidative stress by regulating mitochondrial function.

Our previous research has proven that rhein relieves oxidative stress by activating the SIRT1/PGC-1*α*-regulated mitochondrial biogenesis *in vitro* [29]. Thus, western blot was employed to detect the protein expression of SIRT1, PGC-1*α*, and NRF1 in the hippocampal tissues. As illustrated in Figures 6(c) and 6(d), compared with the WT group, a notable decrease in the expression of SIRT1, PGC-1*α*, and NRF1 existed in the APP/PS1 group, manifesting the impaired mitochondrial biogenesis. Nevertheless, rhein treatment remarkably increased the expression of SIRT1, PGC-1*α*, and NRF1. Therefore, it was noted that rhein could promote mitochondrial biogenesis as an endogenous antioxidant defense system to resist oxidative stress.

While mitochondrial biogenesis contributes to the mass of mitochondria, mitochondrial fission increases the actual number of mitochondria, and mitochondrial fusion controls the actual quality of mitochondria [42]. It is widely believed that abnormal mitochondrial dynamics occurs in AD progression [43–45]. Damaged mitochondria get into constant fission, yet healthy mitochondria cannot restore them by a fusion process, leading to numerous populations of damaged mitochondria and long-term oxidative stress, thus neuronal injury. To determine the mitochondrial dynamics, DRP1, a marker for mitochondria in fission, and MFN1, a marker for mitochondria in fusion, were analyzed by western blotting, respectively. Figures 6(e) and 6(f) show that DRP1 in APP/PS1 was significantly increased, whereas MFN1 was decreased, which was consistent with previous studies. Dramatically, rhein treatment balanced the mitochondrial dynamics from fission toward fusion by downregulating the expression of DRP1 and upregulating that of MFN1. It could be found that rhein promoted mitochondrial biogenesis and consequently balanced mitochondrial dynamics. Overall, the above results confirmed that promotion of the SIRT1/PGC-1*α*-regulated mitochondrial biogenesis for relieving oxidative stress was the key mechanism of rhein for the treatment of AD.

## 4. Discussion

A growing body of evidence demonstrates that neuronal oxidative stress contributes significantly to the pathogenesis and progression of AD, and activating the endogenous antioxidant pathway represents a great promising target to modulate AD pathology [46, 47]. Previous research conducted in our group unveils that rhein effectively relieves mitochondrial oxidative stress in primary neurons induced by A*β*_42_ oligomers, and mitochondrial biogenesis regulated by the SIRT1/PGC-1*α* pathway is a potential antioxidant pathway involved [29]. While impressive *in vitro* antioxidant activity and neuroprotection were obtained, pharmacodynamics evaluation of rhein for AD *in vivo* still needs to be carried out. Therefore, the objective of the current study is to evaluate the effectiveness of rhein for the treatment of AD on an APP/PS1 mouse model and explore its therapeutic mechanisms.

Cognitive impairment is a characteristic feature of AD. Nesting and NOR tests were employed to evaluate cognitive function, exploration, and memory abilities, respectively. It was found that cognitive and memory abilities were declined in APP/PS1 mice. Fortunately, they were improved by rhein after a one-month treatment. Inspired by the positive effects on the behavior tests, a pathological examination in the brain of APP/PS1 mice was further performed. Fortunately, rhein treatment largely reduced the A*β* burden in the brain of APP/PS1 mice as well as neuroinflammation. Meanwhile, these results were confirmed by using ELISA kits. Eventually, the regulation of the above pathological phenotypes supported the results of behavior tests. Our results strongly suggested the ability of rhein to alleviate the pathology of AD.

Natural antioxidants in the brain, such as CAT, GSH-Px, and GSH, serve as a first-line defense mechanism [48]. CAT and GSH are vital antioxidants against hydrogen peroxides and hydroperoxides. GPH-Px detoxifies peroxides by reacting with GSH and converting it into GSSG [49], whereas, relative to other organs, these endogenous antioxidants are less abundant in the brain [3, 50]. Thus, in the state of oxidative stress in AD, they are prone to reach excessive consumption. Ultimately, central neurons cannot defend against oxidative stress by these limited natural antioxidants, resulting in neural damage and apoptosis. In the hippocampus of APP/PS1 mice, decreased activities of CAT and GSH-Px as well as low GSH content were detected. Additionally, the increased levels of GSSG and MDA, the oxidative damage biomarkers, confirmed the occurrence of oxidative stress in the brain. Interestingly, rhein treatment reversed these pathological indicators. The above results testified that rhein had the ability to relieve oxidative stress *in vivo*.

The integrality of synapses plays an important role in neurons for receiving, processing, and exchanging information in a complex neural network, which is the basis for learning and memory of brain function [51, 52]. In AD, neuronal oxidative damage is considered a key component and leads to a decline in learning and memory of brain function [53]. Impressively, rhein restored the density and number of neurons in APP/PS1 mice, which was connected with the inhibition of the caspase-3-related apoptosis cascade activated by cytochrome c, an apoptosis factor from damaged mitochondria. It was concluded that rhein could protect neurons from apoptosis by relieving oxidative stress.

It has been reported that mitochondrial dysfunction occurs in the pathology of AD [16, 41]. ETC on the mitochondrial inner membrane destroyed by toxic A*β* leads to decreased activities of enzymes in ETC complexes. It was consistent with the decreased activity of CytOx in the present study. Additionally, the decreased SOD detected in the study indicates that mitochondria cannot eliminate excess ROS. Fortunately, the activities of mitochondrial CytOx and SOD were recovered after treatment with rhein. Thus, it was assumed that rhein has a positive effect on the improvement of mitochondrial function.

Mitochondrial biogenesis as an endogenous antioxidative defense system facilitates the generation of healthy mitochondria to supply abundant antioxidant enzymes, mainly SOD, when there is a high demand against oxidative stress [54]. Meanwhile, mitochondrial biogenesis endows mitochondria the ability to deal with oxidatively damaged mitochondria [23, 55]. That is to say, the newly generated healthy mitochondria tend to repair the damaged ones by mitochondrial fusion to maintain their morphology, distribution, and function. The SIRT1/PGC-1*α* pathway is of great significance for the regulation of mitochondrial biogenesis. SIRT1 interacts with and activates PGC-1*α* by deacetylation [56, 57]. PGC-1*α* is considered to play a role in the control of mitochondrial biogenesis through transcriptional regulation. Deacetylated PGC-1*α* acts on transcription factors, such as NRF1, which is in charge of transcriptional activation of mtDNA in the nucleus, leading to synthesizing new mitochondria with healthy functions [58]. Based on our previous *in vitro* antioxidant mechanisms of rhein, the expression of proteins related to mitochondrial biogenesis in the hippocampus of APP/PS1 mice was analyzed by western blotting. As expected, impaired mitochondrial biogenesis was proven by downregulated SIRT1, PGC-1*α*, and NRF1. After treatment with rhein, the expression levels of SIRT1 and PGC-1*α* were remarkably upregulated together with NRF1. Thus, it could be declared that rhein improved mitochondrial biogenesis in AD mice.

The mitochondrial dynamics is highly dependent on mitochondrial biogenesis to control the number of healthy mitochondria by a fission/fusion process [59, 60]. Mitochondria are affected by mitochondrial fission/fusion proteins [61, 62]. Mitochondrial fission proteins like DRP1 contribute to regulating the number of mitochondria, and fusion proteins like MFN1 help to assure the quality of mitochondria. Recent studies have shown that there is an imbalance in DRP1/MFN1 in AD progression. To elucidate the mechanism underlying the reparation of damaged mitochondria by mitochondrial biogenesis, DRP1 and MFN1 were estimated by western blotting, respectively. Indeed, compared with WT mice, abnormal mitochondrial dynamics occurred with increased expression of DRP1 and decreased expression of MFN1 in the hippocampus of APP/PS1 mice, resulting in the proliferation of damaged mitochondria. Thanks to the improved mitochondrial biogenesis by rhein, newly generated healthy mitochondria expressed the decreased expression of DRP1 and increased expression of MFN1 to a normal level, indicating the recovery of mitochondrial dynamics to repair damaged mitochondria and inhibit the production of ROS from ETC.

Mounting evidence has shown that ROS accelerate the generation and accumulation of misfolded A*β* by upregulating the expression level of amyloid precursor protein in neurons as well as its active enzyme, *β*-site APP-cleaving enzyme 1 [63, 64]. Inspired by the decreased A*β* burden in the brain of rhein-treated APP/PS1 mice, we wonder whether rhein has a positive effect on the aggregation and disaggregation of A*β*. Regrettably, through the cell viability assay and circular dichroism spectroscopy experiments, no significant effect was observed to support the direct regulation of rhein on A*β* (Supplementary Figures 1(a)–1(d)). Thus, we speculate that rhein could intervene with the dynamic forming process of A*β* by relieving oxidative stress. An in-depth study will be carried out in our group in the future.

## 5. Conclusions

Based on our previous research, the therapeutic effect and mechanisms underlying relieving oxidative stress of rhein for AD were further investigated on APP/PS1 mice here. As a result, rhein could permeate into the brain, reduce A*β* deposits and neuroinflammation, and finally ameliorate cognitive impairment in APP/PS1 mice. Furthermore, rhein significantly reversed neuronal apoptosis by its surprising antioxidant ability. Impressively, we revealed that rhein activated the SIRT1/PGC-1*α* pathway to improve mitochondrial biogenesis, which worked as an endogenous antioxidant defense system. Mitochondrial biogenesis could not only increase the endogenous antioxidant enzymes to scavenge excess ROS but repair damaged mitochondria by correcting mitochondrial dynamics to inhibit the production of ROS from ETC, synergistically alleviating neuronal oxidative stress. Taken together, the antioxidant mechanism of rhein reflects that the regulation of upstream mitochondrial biogenesis might have an effective therapeutic potential for AD. Our research expands the horizon of improving mitochondrial biogenesis to relieve oxidative stress for the treatment of AD.

## Figures and Tables

**Table 1:** Main pharmacokinetic parameters of rhein in rat plasma after a single intravenous injection with rhein (*n* = 5, mean ± SD).

Parameters	Value
*t*_1/2_ (h)	8.09 ± 1.23
AUC_0‐*t*_ (*μ*g·h/mL)	60.42 ± 8.17
AUC_0‐∞_ (*μ*g·h/mL)	61.37 ± 7.97
MRT (h)	1.47 ± 0.22
*V*_d_ (L/kg)	1.95 ± 0.29
CL (L/h/kg)	0.16 ± 0.02

## Data Availability

The data used to support the findings of this study are available from the corresponding authors upon request.

## Abbreviations

A*β*: *β*-Amyloid
AD: Alzheimer's disease
AUC: Area under the plasma concentration-time curve
BBB: Blood-brain barrier
BCA: Bicinchoninic acid
CAT: Catalase
CL: Clearance
CytOx: Cytochrome C oxidase
DRP1: Dynamin-related protein 1
ETC: Electron transport chain
GSH: Glutathione
GSH-Px: Glutathione peroxidase
GSSG: Oxidized glutathione
IL-1*β*: Interleukin-1*β*
MDA: Malondialdehyde
MFN1: Mitofusin 1
MRT: Mean residence time
mtDNA: Mitochondrial DNA
NOR: Novel object recognition
NRF1: Nuclear respiratory factor 1
PGC-1*α*: Peroxisome proliferator-activated receptor gamma coactivator 1-alpha
ROS: Reactive oxygen species
SD: Sprague-Dawley
SIRT1: Sirtuin 1
SOD: Superoxide dismutase
*t*_1/2_: Elimination half-life
TNF-*α*: Tumor necrosis factor-*α*
*V*_d_: Apparent volume of distribution
WT: Wild type.

## Supplementary Materials

Supplementary Materials Supplementary materials and methods. 1.1: sample processing. 1.2: UPLC-TQ-MS/MS method. 1.3: preparation of A*β*_42_ solutions. 1.4: primary neuron culture and treatment. 1.5: cell viability assay. 1.6: circular dichroism (CD) spectroscopy assay. Supplementary Figure 1: the therapeutic effect of rhein on APP/PS1 mice was not involved in A*β* aggregation or disaggregation.
