# Supplementary material for: Rhein Ameliorates Cognitive Impairment in an APP/PS1 Transgenic Mouse Model of Alzheimer's Disease by Relieving Oxidative Stress through Activating the SIRT1/PGC-1α Pathway
Source: Oxid Med Cell Longev. 2022 Mar 22;2022:2524832. doi: 10.1155/2022/2524832 (PMC8964225; doi:10.1155/2022/2524832)
Supplement: Supplementary Materials — and methods. 1.1: sample processing. 1.2: UPLC-TQ-MS/MS method. 1.3: preparation of Aβ42 solutions. 1.4: primary neuron culture and treatment. 1.5: cell viability assay. 1.6: circular dichroism (CD) spectroscopy assay. Supplementary Figure 1: the therapeutic effect of rhein on APP/PS1 mice was not involved in Aβ aggregation or disaggregation. [file 2524832.f1.doc]

**Supplementary materials**

**Rhein** **Ameliorates Cognitive Impairment in an APP/PS1 Transgenic Mouse Model of** **Alzheimer’s Disease by Relieving Oxidative Stress through Activating the SIRT1/PGC-1α Pathway**

Zhihui Yin, Demin Gao, Ke Du, Chen Han, Yuhan Liu, Ying Wang^*^ and Xiaoyan Gao^*^

School of Chinese Materia Medica, Beijing University of Chinese Medicine, Beijing 102488, China

Correspondence should be addressed to Ying Wang; wangy174@126.com and Xiaoyan Gao; gaoxiaoyan@bucm.edu.cn

**Supplementary Materials and Methods**

*1.1. Sample processing*.

Each 10 μL plasma was mixed with 20 μL HCl (2 M) and 1 mL ethyl acetate (containing 2 μg/mL 1,8-dihydroxyanthraquinone as internal standard), and each 200 μL brain homogenate was mixed with 20 μL HCl (2 M) and 1 mL ethyl acetate (containing 0.2 μg/mL 1,8-dihydroxyanthraquinone as internal standard) to extract rhein. After centrifugation at 4°C (3500 rpm, 10 min), 800 μL of the upper layer solution was collected and dried by vacuum centrifugal concentrator. Following redissolved with 100 μL methanol and centrifuged at 4°C (16500 × g, 15 min), 2 μL supernatant was injected into the ultra-performance liquid chromatography coupled with triple quadrupole mass spectrometry (UPLC-TQ-MS/MS) system for analysis.

*1.2. UPLC-TQ-MS/MS method*.

UPLC-TQ-MS/MS method was performed on Waters ACQUITY^TM^ Ⅰ-Class (Waters Corporation, Milford, MA, USA) coupled online to a Waters Xevo TQ-S Triple Quad Mass Spectrometer. Chromatographic separation was carried out using an ACQUITY UPLC HSS T3 column (2.1 × 100 mm, 1.8 μm). The column was maintained at 40°C, and a gradient of 0.1% formic acid in water (solvent A) and acetonitrile (solvent B) was used as follows: 0-0.1 min, 40% B; 0.1-1.5 min, 40-100% B; 1.5-3 min, 100% B; 3-3.1 min, 100-40% B; 3.1-5.5 min, 5% B. The flow rate was 0.4 mL/min.

TQ-MS/MS analysis was operated in negative ion mode under the following conditions: capillary voltage, 2500 V; source temperature, 150°C; desolvation temperature, 500°C; desolvation gas flow, 1000 L/h, cone gas flow, 50 L/h. Quantification was performed using multiple reaction monitoring (MRM), with transitions of *m/z* 283.00→183.03 (cone voltage of 10 V and collision energy of 28 eV) for rhein, and *m/z* 239.01→211.04 (cone voltage of 62 V and collision energy of 20 eV) for internal standard. Data were obtained and analyzed by MassLynx software.

*1.3. Preparation of Aβ_42_ solutions*.

The Aβ_42_ monomers were prepared at first. The lyophilized Aβ_42_ peptides (CAS: 107761-42-2, Nanjing Peptide Biotech Co., Ltd., Nanjing, China) were dissolved in 1,1,1,3,3,3-hexafluoro-2-propanol (HFIP) and kept shaking for 3 h at 4°C. And then, HFIP was removed under a gentle N_2_ stream and the films were formed. Thereafter, the films were dissolved with sodium hydroxide, and the Aβ_42_ monomers were collected after centrifugation at 4°C (16000 × g, 10 min). To prepare Aβ_42_ oligomers (Aβ_42_O), 50 μM Aβ_42_ monomers were incubated with shaking at 150 rpm for 24 h. To prepare Aβ_42_ fibrils, 50 μM Aβ_42_ monomers were incubated with shaking at 150 rpm for 4 d.

For Aβ_42_ aggregation study, Aβ_42_ monomers were incubated with rhein at three doses of 50, 100, and 200 μM for 24 h at 37°C, respectively. For Aβ_42_ disaggregation study, Aβ_42_ fibrils were incubated with rhein at three doses of 50, 100, and 200 μM for 7 d at 37°C, respectively.

*1.4. Primary neuron culture and treatment*.

Primary neurons were extracted from neonatal SD rats as described previously [1]. Isolated primary neurons with a cell density at 5 × 10^5^ cells/mL were seeding in poly-L-lysine precoated 96-well and cultured in a humidified atmosphere containing 5% CO_2_. After 7 days of culture, neurons were used for follow-up experiments.

To examine the cytotoxicity of Aβ_42_O pretreated with or without rhein, Aβ_42_ monomers, Aβ_42_O, Aβ_42_ pretreated with different doses of rhein (50, 250, and 500 μM) were diluted ten times to a final Aβ_42_ concentration of 5 μM and incubated with primary neurons for 24 h, respectively. Meanwhile, the control group was treated with the same blank medium.

*1.5. Cell viability assay*.

After being incubated with Aβ_42_O pretreated with or without rhein, cell viability was measured by the MTT assay. In brief, 100 μL MTT (0.5 mg/mL) was added and incubated for 4 h. Then, 100 μL DMSO was added after the solution was removed. Finally, the absorbance at 490 nm was measured using an Epoch Microplate Spectrophotometer (BioTek Instruments Inc., Winooski, VT, USA).

*1.6. Circular dichroism (CD) spectra assay*.

The prepared Aβ_42_ in 100 μL was added to a 0.5 mm path length quartz cell for measurements (195 to 260 nm). Each spectrum was scanned three times at a speed of 1 nm/s using a Chirascan spectrometer (Applied Photophysics Ltd., Surrey, BA, UK).

**Supplementary figures**


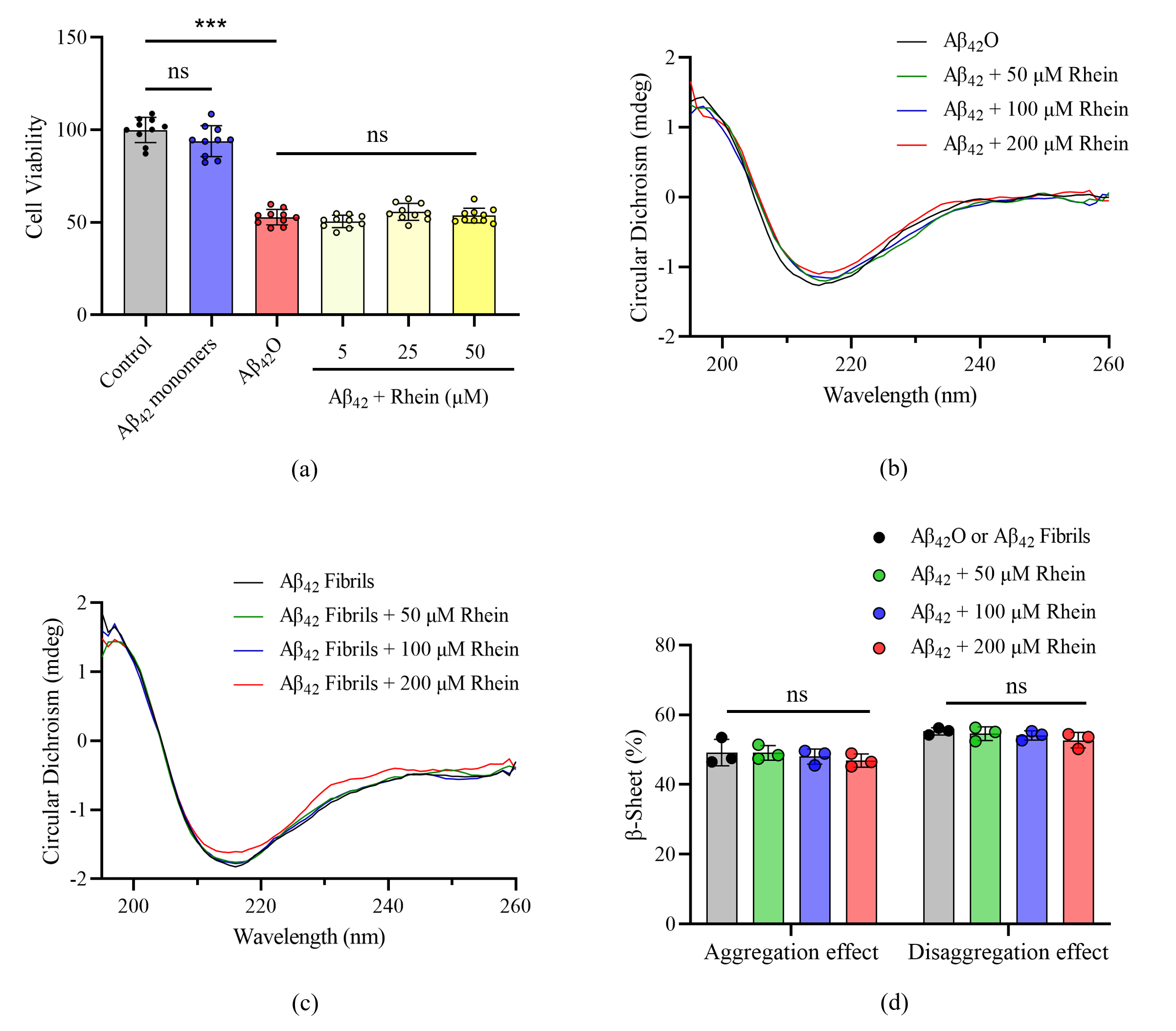


**Supplementary Figure 1**. The therapeutic effect of rhein for APP/PS1 mice did not involve in Aβ aggregation or disaggregation. For Aβ_42_ aggregation study, rhein at 50, 100, and 200 μM were incubated with 50 μM Aβ_42_ monomers for 24 h, respectively. For Aβ_42_ disaggregation study, rhein at 50, 100, and 200 μM were incubated with 50 μM Aβ_42_ fibrils for 7 d, respectively. (a) Cell viabilities of primary neurons were measured by the MTT assay after incubated with Aβ_42_ in the absence or presence of rhein for 24 h. In this part of the experiment, Aβ_42_ monomers with or without rhein were pre-incubated for 24 h in PBS before cell administration. Meanwhile, untreated primary neurons were the control group. (b, c) The effects of rhein on Aβ_42_ aggregation or disaggregation were monitored by circular dichroism (CD) spectra. (d) Quantitative analysis of the proportions of β-sheet. The results are expressed as the mean ± standard deviation (SD). ****p* < 0.001 and ns, not significant.

## References

[1] Y. T. Dong, K. Cao, L. C. Tan et al., "Stimulation of SIRT1 attenuates the level of oxidative stress in the brains of APP/PS1 double transgenic mice and in primary neurons exposed to oligomers of the amyloid-beta peptide,*" Journal of Alzheimer's Disease*, vol. 63, no. 1, pp. 283-301, 2018.
